# Studyholism and Study Engagement in Adolescence: The Role of Social Anxiety and Interpretation Bias as Antecedents

**DOI:** 10.3390/ijerph19095261

**Published:** 2022-04-26

**Authors:** Yura Loscalzo, Marco Giannini

**Affiliations:** Department of Health Sciences, School of Psychology, University of Florence, 50135 Florence, Italy; marco.giannini@unifi.it

**Keywords:** Studyholism, study engagement, study addiction, Workaholism, work addiction, work engagement, OCD, social anxiety, interpretation bias, interpretative bias

## Abstract

Studyholism (or obsession toward study) is a new potential clinical condition that, in contrast with Study Engagement, is associated with negative outcomes. However, previous studies showed that both Studyholism and Study Engagement predict social impairment due to study. Therefore, we analyzed the role of social anxiety and interpretation bias as predictors of Studyholism and Study Engagement in 541 adolescents (*M*_age_ = 16.30 ± 1.59; 66% girls). We performed a path analysis model, MANOVAs, and Mann–Whitney tests. Among the main findings, social anxiety is a positive predictor of both Studyholism and Study Engagement. Hence, this provides further support to the conceptualization of Studyholism as an OCD-related disorder (or as an internalizing disorder) and suggests the need of screening socially anxious adolescents for the presence of Studyholism and engaged students for the presence of high social anxiety. Moreover, Studyholism is predicted by a negative interpretation style in non-social situations, while a positive interpretation style predicts Study Engagement in social and non-social situations. Hence, Studyholism and social anxiety are two different diagnoses, even if social anxiety might fuel Studyholism. Moreover, interventions to reduce Studyholism should decrease the tendency to interpret non-social situations negatively or neutrally.

## 1. Introduction

Studyholism is a term introduced in the literature by Loscalzo and Giannini [1] to define a new potential clinical condition associated with problematic overstudying and that they suggested being more similar to an obsession than to an addiction toward study. Loscalzo and Giannini [2] advocated using “problematic overstudying” when referring to this problem behavior regardless of a specific theorization. Studyholism, instead, refers to Loscalzo and Giannini’s [1] conceptualization of problematic overstudying as an Obsessive-Compulsive (OCD) related disorder, while Study Addiction [3] conceptualizes it as a behavioral addiction characterized by the seven core components of substance addiction.

Loscalzo and Giannini [1] suggested going beyond the addiction framework, as highlighted by Kardefelt-Winther [4], and considered the possibility that problematic overstudying is made up of both addiction and obsessive symptoms. Moreover, they suggested that either high or low levels of Study Engagement could be associated with problematic overstudying (hence distinguishing two subtypes of Studyholic: Engaged and Disengaged Studyholics). Then, the growing evidence gathered through their cross-sectional studies suggested that the conceptualization of Studyholism as an OCD-related disorder might be more appropriate than the theorization of problematic overstudying as a behavioral addiction or as a condition including both addiction and obsessive symptoms.

The first data have been gathered to create an instrument for evaluating Studyholism and Study Engagement; namely, the Studyholism Inventory (SI-10) [5,6]. Loscalzo et al. [5] developed a pool of 68 items covering addiction symptoms, obsessive symptoms, and study engagement features to create this scale. However, the Explorative Factor Analysis conducted on Italian college students suggested the deletion of all the addiction items from the final version of the SI-10. Therefore, Loscalzo and Giannini [1] reviewed their theory and suggested that Studyholism is made up of obsessive-compulsive symptoms only (and either high or low levels of Study Engagement). Then, Loscalzo and Giannini [2] substantiated their theorization of Studyholism as a new potential OCD-related disorder through a thoughtful comparison of DSM-5 [7] criteria for OCD, substance-use disorder, and obsessive-compulsive personality disorder. However, the authors were willing to deepen the analysis of Studyholism through research that could help shed light on the internalizing and/or externalizing nature of problematic overstudying.

Hence, they tested many of the potential antecedents and outcomes of Studyholism (as listed in their theoretical model [1]) on both youth [8] and adolescent [9] students. These studies supported their conceptualization of Studyholism as an OCD-related disorder or, more generally, an internalizing (rather than externalizing) disorder since they found that trait worry is a strong predictor of Studyholism in both the student samples. Worry is indeed an internalizing feature that contributes to OCD [10]. Next, two other studies supported the OCD-related conceptualization. Loscalzo and Giannini’s [11] study mainly focused on the impact of the COVID-19 pandemic (and the related lockdown) on college students’ academic path and attitudes toward studying, including Studyholism and Study Engagement. However, it also showed that intolerance for uncertainty, a feature of internalizing disorders, including OCD [12,13,14], predicts Studyholism. Moreover, Loscalzo and Giannini’s [15] study highlighted that Studyholism is positively predicted by internalizing symptoms (obsessive-compulsive, depression, and anxiety symptoms) and negatively predicted by externalizing variables (psychoticism and boredom susceptibility).

In sum, the literature concerning Studyholism has been rising in the last few years, and more evidence has been presented relating to the conceptualization of Studyholism as a new potential OCD-related disorder or, more generally, an internalizing disorder. However, as Loscalzo and Giannini have pointed out in their papers, e.g., [2,8,15,16], the literature on problematic overstudying is still too scant to reach any firm conclusions, and further studies are needed. In line with this, Loscalzo and Giannini [17] suggested their Studyholism DSM-like criteria as being tentative. Therefore, they proposed them as a valuable frame of reference for future studies addressing this construct, but they stressed that quantitative and qualitative studies are needed for analyzing which criteria need to be deleted (or modified) and which need to be added based on new research evidence. In sum, the criteria stated that Studyholism is characterized by persistent and recurrent problematic studying behaviors that lead to clinically significant impairment or distress. Moreover, the students had study-related obsessions and/or study-related compulsions over the last six months. The criteria also comprehend the usual DSM-5 [7] exclusion criterion: physiological effects of a substance or medical condition and other mental disorders. Loscalzo and Giannini [17], based on their first empirical evidence, also included these two specifiers: (i) study engagement level (high, average, low); (ii) main area of impairment (academic, social, both areas).

Even if Studyholism is an emerging construct in the scientific literature, it is critical to analyze it further as it is widespread in Italian youths [18] and adolescents [19]. Moreover, Loscalzo and Giannini [8] and Loscalzo [9] highlighted several downsides associated with Studyholism in youths and adolescents, including higher school dropout intention and physical and psychological impairment. Study Engagement, instead, proved to be a protective factor since it is associated with lower school dropout intention, higher academic performance, and better physical and psychological functioning. Therefore, the current study aims to address new potential antecedents of Studyholism, aiming to detect those factors which might be addressed to reduce Studyholism and its related functional impairment. Until now, just a few antecedents have been proven to be useful targets for the prevention of Studyholism: trait worry [8,9] and, for adolescents only, the overstudy climate spread by teachers, even if this is a weak predictor [9]. Hence, it is vital to detect other psychological variables to be addressed to foster students’ well-being and reduce Studyholism.

### 1.1. The Present Study

Even with a few differences, Studyholism and Study Engagement are both associated with higher time spent studying and social impairment due to study behaviors; that is, with an adverse outcome concerning social well-being [8,9]. Therefore, we decided to extend the findings of these studies [8,9] by focusing the current research on social variables, which might be targeted in both Studyholics and Engaged students to prevent the social impairment associated with their high investment of time and energy in studying. Hence, we selected social anxiety and interpretation bias as social-related variables to be analyzed as antecedents of Studyholism and Study Engagement.

The interpretation bias is, in fact, a contributing factor to social anxiety in adolescents [20,21,22,23,24,25]. Castillo and Leandro [26] defined interpretation bias as the tendency to systematically assign a threatening meaning to an objectively ambiguous stimulus, which has several possible interpretations. An example of an ambiguous stimulus (concerning social anxiety) might be a person yawning during a speech. The one who is talking cannot know the reason for the yawn; therefore, this is an ambiguous stimulus that requires interpretation. The person might interpret the stimulus negatively, positively, or neutrally. If the person explains the yawn as due to the speech being boring (i.e., the person selects a negative interpretation), he/she uses a negative interpretation of the ambiguous stimulus. If this person usually chooses the negative interpretation (instead of a positive or neutral one) for different ambiguous stimuli, he/she is characterized by a negative interpretation bias.

In the context of social anxiety, as highlighted by Amir and Bomyea [27], this cognitive bias deserves to be studied since social interactions are generally ambiguous. Consequently, the tendency to systematically interpret ambiguous social situations negatively might be associated with a higher risk for social anxiety. We speculate that the interpretation bias might have a role also in Studyholism and Study Engagement. Studying might take place in a “private” form (e.g., when the student is preparing him/herself for a written or oral test). However, it also foresees a “public” form (or social form), such as when students prepare together for a test or when they are in front of the teacher and/or the class for being interviewed or judged for their school performance. Finally, receiving a bad mark on a test might be classified as a non-social situation. However, it might be relevant in the context of study attitudes since it might give room to various types of interpretation, such as: “I am not good at this study, I will give up” (negative interpretation), “I was prepared, but the teachers asked too many difficult questions and did not give me enough time. I will recover my grade with the next test” (positive interpretation), and “I was tired. It might happen to have a lower grade than usual” (neutral interpretation).

#### Aim and Hypotheses

In sum, we aim to evaluate whether social anxiety and interpretation bias, either in social and non-social situations, are predictors of Studyholism and Study Engagement. For evaluating the interpretation bias, we selected a scale that allows measuring eight variables, four for each type of situation (social and non-social situations): the tendency to interpret ambiguous situations positively, negatively, and neutrally, and the tendency to believe in negative interpretations. Moreover, we aim to analyze whether Studyholism and Study Engagement levels differ based on the type of school (professional school and high school) and school year.

Based on the previous literature, we hypothesize: (i) social anxiety, as an internalizing feature, is a good positive predictor of Studyholism ([1,8,9,15]; for Study Addiction, [28]); (ii) social anxiety is a positive predictor of Study Engagement since, based on the findings suggesting that Study Engagement is associated with social impairment due to study [8,9], we speculate that this type of heavy study investment might be a coping strategy for social anxiety symptoms (in line with Loscalzo and Giannini’s [15] study, which found that Study Engagement is positively predicted by anxiety and paranoid symptoms, and also similarly to work engagement, which seems to be a coping strategy for somatic symptoms [29]); (iii) the eight variables related to the interpretation bias in social and non-social situations might play a role as predictors of Studyholism and Study Engagement; however, since this is the first study addressing these relationships, we did not posit specific hypotheses for each variable, except for a general expectation of a more positive style of interpretation for Study Engagement and a more negative interpretation style for Studyholism. Finally, we aim to explore the differences between students with high and low levels of Studyholism and Study Engagement on the variables analyzed, and between the two types of Studyholic, in line with previous studies [8,9,15]. However, we do not posit specific hypotheses since this is the first study addressing social anxiety and interpretation bias regarding Studyholism and Study Engagement. Moreover, we will explore whether Studyholism and Study Engagement levels differ based on the type of school and school year, as hypothesized by Loscalzo and Giannini [1], but not fully supported by the few studies about Studyholism in adolescents [9,19].

## 2. Materials and Methods

### 2.1. Participants

We had the participation of 541 Italian adolescents from Central Italy and aged between 13 and 20 years (*M*_age_ = 16.30 ± 1.59; 66% girls). The students attended a professional school (culinary art and hotel management school, 52.3%) or a high school (47%). More specifically, the percentage of students attending classical, socio-economic, and language high school were 3.9%, 21.8%, and 21.3%. There are few missing data concerning the type of school attended (0.7%). All the five school years were represented; the proportions of students in years 1 to 5 were 18.7%, 17.9%, 25.7%, 21.8%, and 15.9%.

Concerning school-related variables, most participants declared that they usually study on the weekend (65.1%), and a minority said that they have repeated at least a school year (22.6%), in line with the presence of students aged more than 19 in our sample. Italian grades range between 0 and 10, with 6 being the sufficiency and scores between 0 and 5 representing different insufficiency levels. The participants’ Grade Point Average range between 3 and 9, with a mean of 6.96 ± 0.91. Finally, concerning the time spent studying, the hours of study per day (generally) range between 0 and 10 (*M* = 1.90 ± 1.28), while the hours of study before a written or oral school test range between 0 and 8 (*M* = 2.38 ± 1.46). For the days of study per week, the range is between 0 and 7 (*M* = 3.90 ± 1.94).

### 2.2. Materials

#### 2.2.1. Studyholism Inventory (SI-10) 

The SI-10 [6] is a self-report scale that allows evaluating Studyholism (e.g., I cannot relax because of worries about studying) and Study Engagement (e.g., I do the very best I can to get the best grades) through four items (plus a filler item) per scale. It also comprehends a head-sheet with questions about study habits, such as studying on the weekend, time spent studying, and Grade Point Average. The response format is a 5-point Likert scale ranging between 1 (Strongly Disagree) to 5 (Strongly Agree). The SI-10 is currently available in Italian, Polish, Croatian, Spanish, Indonesian, and English. For the present study, we used the Italian version [5,6], which proved to have good psychometric properties on Italian adolescents [19]. More specifically, the α values in the adolescent sample are 0.78 (Studyholism) and 0.79 (Study Engagement) [19].

#### 2.2.2. Social Phobia Inventory (SPIN) 

The SPIN [30] is a 17-item self-report scale that evaluates social anxiety through the total score of its 17 items (e.g., sweating in front of others provokes me anxiety; I avoid talking to anyone who represents authority). The response format is a 5-point Likert scale ranging between 0 (Not at all) and 4 (Extremely). The Italian version has good internal reliability, as Cronbach’s alpha is 0.87 [31]. Even if the SPIN was created for adults, many studies in the literature used it with adolescents [32,33,34,35,36], including Italian studies [21,25].

#### 2.2.3. Adolescents’ Interpretation and Belief Questionnaire (AIBQ) 

The AIBQ [20] is a questionnaire designed for measuring interpretation bias in adolescents. More specifically, it consists of five ambiguous social situations (e.g., give a presentation in class) and five ambiguous non-social situations (e.g., being unable to find the bike locked up somewhere). Each situation ends with a question highlighting the ambiguity of the scenario and asking what the explanation could be for what happened in the scene. Next, there are listed three interpretations: positive, negative, and neutral (different order in different questions), and the participant indicates for each interpretation how likely each of them could pop un in his/her mind, using a scale ranging from 1 (does not pop up in my mind) to 5 (definitely pops up in my mind). Finally, all the interpretations are presented again, and the participant selects the one which is the most believable for him/her. The scoring of this last section foresees to give 1 point for positive interpretation, 2 points for neutral interpretation, and 3 points for negative interpretation. In sum, the AIBQ allows scoring the following scales: (i) negative interpretations in social situations; (ii) neutral interpretations in social situations; (iii) positive interpretations in social situations; (iv) negative interpretations in non-social situations; (v) neutral interpretations in non-social situations; (vi) positive interpretations in non-social situations; (vii) belief in negative interpretations in social situations; and (viii) belief in negative interpretations in non-social situations. We administered the Italian translation of the AIBQ [37]. The alpha values are not satisfactory [37]; however, this is the only instrument available in the literature for evaluating the interpretation bias in adolescents, and it has been previously used in other Italian studies [21,25].

### 2.3. Procedure

First, we got the study approval from the Ethical Committee of the University of Florence. Next, we asked for the written authorization to collect data from the Head of a school comprehending more curricula (three types of high school and a professional school) aiming to have students from different school backgrounds. Next (after collecting the informed consent, signed by the participants and their parents), the questionnaire was administered during school hours: the students filled it on the computer in the school’s informatic room. The questionnaire compzrehended the instruments previously described and a first section for demographic data (e.g., gender, age).

### 2.4. Data Analysis

We performed the analyses through SPSS.27 (Chicago, IL, USA) and AMOS.20 (Chicago, IL, USA).

First, to perform the Structural Equation Model (SEM), we analyzed the variables’ descriptive statistics (including skewness and kurtosis) and their zero-order correlations. Then, we run the SEM—more specifically a path analysis (Maximum Likelihood estimate method)—with social anxiety and the eight AIBQ variables (i.e., negative, positive, and neutral interpretations, as well as belief in negative interpretations, both in social and non-social situations) as predictors of Studyholism and Study Engagement. To evaluate the fit of the model, we used the cut-off values provided by Byrne [38], Hu and Bentler [39], and Reeve et al. [40].

Next, aiming to evaluate differences between students characterized by high/low levels of Studyholism/Study Engagement, we performed two MANOVAs with social anxiety and AIBQ scales as dependent variables. For evaluating if there is a difference between Disengaged and Engaged Studyholics on these variables, instead, we used Mann-Whitney tests since the frequency of the two types of Studyholic in the current sample required the use of non-parametric analyses. The two types of Studyholic (and high/low levels of Studyholism/Study Engagement) have been created referring to the SI-10 cut-off values for Italian adolescents [19]. Finally, we performed two MANOVAs to evaluate if Studyholism and Study Engagement levels differ based on the type of school (i.e., culinary art and hotel management school, classical high school, socio-economic high school, and language high school) and the school year.

## 3. Results

### 3.1. Social Anxiety and Interpretation Bias as Predictors of Studyholism and Study Engagement

First, aiming to test a path analysis model with social anxiety and AIBQ variables as predictors of Studyholism and Study Engagement, we analyzed the descriptive statistics of the variables, including skewness and kurtosis, to check if the variables were normally distributed. As shown by Table 1, the normality assumption was fulfilled. Then, we calculated the zero-order correlations of the study variables (see Table 2). In sum, correlation values highlight statistically significant correlations between social anxiety and both Studyholism (average correlation) and Study Engagement (low correlation). Moreover, Studyholism has a low positive correlation with all the AIBQ variables, except for positive interpretations in social situations and belief in negative interpretations in non-social situations. Study Engagement correlates (weakly) with all the AIBQ variables except for negative interpretations in social situations and belief in negative interpretations in non-social situations.

The path analysis showed an excellent fit to the data: CFI = 0.998; NFI = 0.996; RMSEA = 0.037 (C.I. 90% = 0.000–0.081); *χ*^2^/df = 1.72, *p* = 0.142. The predictors included in the model (social anxiety and the AIBQ scales) explain the 21.6% of the variance in Studyholism and the 10.7% of the variance in Study Engagement. More specifically, social anxiety is the strongest predictor of both Studyholism (β = 0.40, *p* < 0.001) and Study Engagement (β = 0.20, *p* < 0.001). About the AIBQ scales, Studyholism is positively predicted by the tendency to have negative and neutral interpretations in non-social situations. Study Engagement, instead, is positively predicted by the tendency to have positive interpretations in non-social situations and, negatively, by the tendency to believe in negative interpretations in social situations. All the other AIBQ variables are not statistically significant predictors of Studyholism and Study Engagement. Table 3 shows the standardized path weight (and *p* value) for all the variables included in the model.

### 3.2. Differences in Social Anxiety and Interpretation Bias between Students with Different Levels of Studyholism and Study Engagement

Using the cut-off values for high and low Studyholism/Study Engagement [19] we created four groups of student: high Studyholism (*n* = 72, 13.3%), low Studyholism (*n* = 169, 31.2%), high Study Engagement (*n* = 66, 12.2%), and low Study Engagement (*n* = 182, 33.6%). Next, we performed two MANOVAs to analyze if there are differences in social anxiety and AIBQ variables between students characterized by high and low levels of Studyholism and high and low levels of Study Engagement.

About Studyholism, the multivariate test highlighted a statistically significant effect on social anxiety and AIBQ scales: *F*(9, 231) = 11.32, *p* < 0.001, η^2^ = 0.31. More specifically, follow-up ANOVAs showed statistically significant differences in social anxiety, negative interpretations in social situations, belief in negative interpretations in social situations, and all the types of interpretations in non-social situations (not for belief in negative interpretations). Students with high Studyholism score higher than those with low Studyholism on these variables. Concerning Study Engagement, the multivariate test is again statistically significant: *F*(9, 238) = 3.87, *p* < 0.001, η^2^ = 0.13. Follow-up ANOVAs indicated a statistically significant effect for social anxiety, positive and neutral interpretations in social situations, and all the types of interpretation in non-social situations (not for belief in negative interpretations). Students with high Study Engagement score higher than those with low Study Engagement on these scales. Table 4 shows the results of follow-up ANOVA analyses.

### 3.3. Differences in Social Anxiety and Interpretation Bias between Engaged and Disengaged Studyholics

Using Loscalzo et al. [19]’s cut-off values for screening the four types of student, we defined two groups: Engaged Studyholics (*n* = 31, 5.7%) and Disengaged Studyholics (*n* = 5, 0.9%). About the other two types of student, there are 101 (18.7%) detached students (i.e., low levels of Studyholism and Study Engagement) and 8 (1.5%) engaged students (i.e., high levels of Studyholism and low levels of Study Engagement).

Then, to evaluate differences in social anxiety and interpretation bias between Disengaged and Engaged Studyholics, we performed Mann-Whitney tests (Table 5 shows the results of these analyses). We found only a statistically significant difference between Engaged and Disengaged Studyholics: Engaged Studyholics, in social situations, have a higher tendency for positive interpretations than Disengaged Studyholics. For the other variables, even if the differences are not statistically significant, the median values showed that Disengaged Studyholics have higher levels of social anxiety and a greater tendency for negative interpretations (and a stronger belief in negative interpretations) in both social and non-social situations.

### 3.4. Differences in Studyholism and Study Engagement among Different Types of School and Different School Years

We performed a MANOVA to analyze if there are differences in Studyholism and Study Engagement levels between students attending different types of school: culinary art and hotel management school (*n* = 283), classical high school (*n* = 21), socio-economic high school (*n* = 118), and languages high school (*n* = 115). The multivariate test highlighted a statistically significant effect: *F*(6, 1064) = 15.38, *p* < 0.001, η^2^ = 0.08. More specifically, follow-up ANOVAs showed statistically significant differences for both Studyholism [*F*(3, 533) = 20.36, *p* < 0.001, η^2^ = 0.10] and study Engagement [*F*(3, 533) = 26.56, *p* < 0.001, η^2^ = 0.13]. Bonferroni post-hoc test indicated that students from culinary art and hotel management school have statistically significant (*p* < 0.001) lower levels of both Studyholism (*M* = 8.94 ± 4.10) and Study Engagement (*M* = 10.26 ± 4.30) than students from the three types of high school. The mean values for Studyholism and Study Engagement are, respectively, 12.86 ± 5.10 and 14.24 ± 4.66 for classical high school; 11.91 ± 4.80 and 12.79 ± 4.43 for socio-economic high school; 12.57 ± 4.50 and 13.27 ± 4.17 for languages high school. There are no statistically significant differences in Studyholism and Study Engagement between the three types of high school.

The last MANOVA aimed at analyzing if there are differences on Studyholism and Study Engagement levels based on the school year: first (*n* = 101), second (*n* = 97), third (*n* = 139), fourth (*n* = 118), and fifth (*n* = 86). The multivariate test highlighted a statistically significant effect: *F*(8, 1070) = 2.62, *p* = 0.008, η^2^ = 0.02. However, follow-up ANOVAs showed a statistically significant difference for Studyholism only: *F*(4, 536) = 3.43, *p* = 0.009, η^2^ = 0.03. More specifically, students in their last year have statistically significant higher levels of Studyholism (*M* = 12.13 ± 5.10) than first (*M* = 10.12 ± 4.46, *p* = 0.034), third (*M* = 10.15 ± 4.65, *p* = 0.020), and fourth (*M* = 9.95 ± 4.81, *p* = 0.010) year students.

For second-year students, the mean value for Studyholism is 10.61 ± 4.19. No other differences between school years emerged.

## 4. Discussion

Studyholism, or obsession with study, is a new potential clinical condition that Loscalzo and Giannini—based on their first research evidence [8,9,11,15]—suggested might be conceptualized as an OCD-related disorder or, more generally, as an internalizing (rather than externalizing) condition. Studyholism is associated with several downsides in the academic, physical, and psychological areas, in contrast with Study Engagement, which is associated with better academic performance and well-being [8,9]. However, both Studyholism and Study Engagement, as two forms of heavy study investment, are associated with higher time spent studying and higher social impairment due to study. Therefore, to detect the psychological features that might be addressed to reduce Studyholism and, more generally, the adverse social outcomes that could be present in Studyholics and Engaged students, we aimed to analyze variables related to social aspects. Hence, we selected social anxiety and interpretation bias as potential predictors of Studyholism and Study Engagement, and we analyzed them in a sample of Italian adolescents attending different types of school (professional and high schools).

First, we tested a path analysis model with Studyholism and Study Engagement as outcomes of the following variables: (i) social anxiety; (ii) negative interpretations in social situations; (iii) positive interpretations in social situations; (iv) neutral interpretations in social situations; (iv) belief in negative interpretations in social situations; (v) negative interpretations in non-social situations; (vi) positive interpretations in non-social situations; (vii) neutral interpretations in non-social situations; (viii) belief in negative interpretations in non-social situations.

The results showed that, as hypothesized, social anxiety is a positive predictor of both types of heavy study investment. More specifically, it is a good predictor of Studyholism (*β* = 0.40), supporting its conceptualization as an internalizing (rather than externalizing) disorder. A previous study on college students showed that some internalizing symptoms positively predict Studyholism, while psychoticism and boredom susceptibility (externalizing variables) are negative predictors [15]. Moreover, trait worry predicts Studyholism in youths and adolescents [8,9]. Hence, the present study builds on previous findings and suggests that Studyholism might be better conceptualized as an OCD-related disorder or, more generally, as an internalizing disorder. Furthermore, it provides a crucial addition to Loscalzo and Giannini’s [15] study, which analyzed many internalizing variables, but that did not have a scale for addressing social anxiety specifically. The instrument they used included a scale labeled “Interpersonal Sensitivity.” However, this scale mainly addresses the feeling of being inadequate and inferior to others, while the scale we used in the present study measures typical social anxiety symptoms. Therefore, it is not surprising that, while Interpersonal Sensitivity is not a predictor of Studyholism [15], social anxiety is a good positive predictor.

Concerning Study Engagement, as hypothesized, social anxiety positively predicts also this type of attitude toward studying that, despite being usually associated with positive outcomes (in contrast with Studyholism), it is also associated with higher social impairment due to study [8,9]. It is based on previous studies [8,9] that we hypothesized that social anxiety could be a positive predictor of Study Engagement, also taking into account that Loscalzo and Giannini [15] suggested that some engaged students might use overstudying behavior to cope with anxiety (and paranoid) symptoms, similarly to engaged workers with regards to somatic symptoms [29]. Hence, the current findings highlight the role of social anxiety as a potential factor for the arising of Study Engagement (especially in the form associated with social impairment). Moreover, they provide further evidence for the need for two different conceptualizations for Studyholism/Study Engagement and Workaholism/Work Engagement. There are differences among the constructs that do not consent to straightforwardly transfer the widely analyzed work constructs into the study areas. Therefore, as stated in a previous paper [15], we recommend using the Studyholism Inventory (SI-10) [6] and the Studyholism Inventory—Extended version (SI-15) [17] to analyze problematic overstudying and Study Engagement instead of using the students’ versions (Bergen Study Addiction Scale, BStAS [3] and Utrecht Work Engagement Scale, Short and Student version—UWES-S [41]) of the two broadly used instruments for the measure of problematic overworking and Work Engagement [42,43]. The SI-10 and the SI-15 have been designed from a pool of items related to study behaviors specifically. This is the reason why, in line with our previous study [15], we used the SI-10 instead of the Italian version of the BStAS [44] and the UWES-S [45]. Concerning the BStAS, other reasons for its discarding are that it is based on the addiction framework and that the Italian version showed some psychometric weaknesses.

In terms of the BStAS, it is also interesting to note that previous research conducted on a specific type of student (music academies) and using the BStAS [3]—which is the instrument designed for measuring Study Addiction [3]—found that social anxiety is a positive predictor of Study Addiction (*β* = 0.24, *p* = 0.017) [28]. We speculate that the results by Lawendowski et al. [28], especially when compared with ours, provide further support for the definition of problematic overstudying as an internalizing disorder since, even when measured through a scale designed for evaluating addiction symptoms, it highlights that social anxiety (an internalizing disorder) is a positive predictor. Moreover, the *β* value found by Lawendowski et al. [28] is similar to the one we found for Study Engagement (*β* = 0.24). Hence, we believe that this supports our speculation that the BStAS does not adequately distinguish between Study Addiction and Study Engagement, as highlighted by previous studies [6,17,44]. In our view, it is critical to distinguish between problematic overstudying and Study Engagement since these are two different constructs that, in some students (i.e., Engaged Studyholics), might be co-present at high levels [1], as is also showed by the high value of correlation between Studyholism and Study Engagement in the current sample.

Despite these theoretical implications, there are also noteworthy practical suggestions arising from the results about the role of social anxiety in predicting Studyholism and Study Engagement. First, given that social anxiety has a significant role in predicting Studyholism, we suggest that socially anxious adolescents should be screened for the presence of Studyholism, aiming to avoid the development of high Studyholism levels, which would lead to greater functional impairment. Moreover, students with high levels of Studyholism could be treated through interventions effective in decreasing social anxiety since they could also reduce Studyholism levels. Hence, future studies should test the efficacy of social anxiety treatments in Studyholism, aiming to discover their actual worth in treating (and preventing) Studyholism. In addition, since social anxiety is a predictor of Study Engagement—and considering that Engaged students might have social impairment [8,9]—we recommend screening Engaged students for the presence of impairing social anxiety, especially if they (or their relatives and teachers) report that they are well-motivated and successful students, who, however, have difficulties in making social connections and have poor relationships. These students should be referred to a clinician to properly diagnose (or exclude) a social anxiety disorder (or high social anxiety). Parents and teachers have a critical function as they might notice that, despite their academic success and physical and psychological well-being, they might have serious social issues that require attention (and intervention).

The last hypothesis addressed by the path analysis model concerns the role of the interpretation bias (both in social and non-social situations) in predicting Studyholism and Study Engagement. Even if we posited that some of the eight variables measured through the Adolescents’ Interpretations and Belief Questionnaire (AIBQ) would have been statistically significant predictors, we did not posit specific hypotheses about each component since this is the first study addressing this bias with regards to Studyholism and Study Engagement. We only expected a positive interpretation style for Study Engagement and a negative interpretation style for Studyholism. The results showed, as anticipated, that a negative style of interpretation predicts Studyholism: it is predicted by the tendency to interpret non-social situations negatively (*β* = 0.16, *p* = 0.002) and neutrally (*β* = 0.15, *p* < 0.001). Moreover, a positive style of interpretation predicts Study Engagement: it is predicted by the tendency to interpret non-social situations positively (*β* = 0.17, *p* = 0.002) and, marginally, by the tendency to believe less in negative interpretations in social situations (*β* = −0.11, *p* = 0.049).

These results further support the conceptualization of Study Engagement as a type of heavy study investment generally characterized by positive features, even if it might be associated with social impairment in some students. In fact, we found a positive style of interpretation for both non-social and social situations. Studyholism, instead, in line with its conceptualization as a negative type of heavy study investment (or as a potential new clinical disorder), is characterized by a negative style of interpretation for non-social situations. However, it is not predicted by a negative interpretation bias in social situations, in contrast with previous studies on socially anxious adolescents [20,21,25]. Consequently, we might suggest that social anxiety, even if a predictor of Studyholism, is a different clinical diagnosis than Studyholism. Therefore, as Loscalzo and Giannini [17] stressed in their DSM-5 tentative criteria, whenever other established clinical diagnoses (including social anxiety disorder) might explain problematic overstudying, a diagnosis of Studyholism should not be used. However, it is valuable to have the option of Studyholism as another diagnosis differentiated by other internalizing disorders.

From a practical perspective, the results on the interpretation bias suggest that for reducing Studyholism, clinicians should address the interpretation style for non-social situations to decrease the tendency to interpret them negatively or neutrally. Therefore, preventive and clinical interventions should decrease negative and neutral interpretations or increase positive interpretations to reach this aim. Since one of the five non-social situations depicted by the AIBQ concerns having received a bad mark on the last school test, we recommend that interventions address the interpretation bias focusing on school situations, particularly school performance. Moreover, it would be interesting to deepen the analysis of the interpretation bias in Studyholism and Study Engagement using a questionnaire designed to tap performance-related ambiguous (social and non-social) situations, to which students might be more sensitive. Finally, based on the current findings, it seems that the interpretation bias is not a proper target for the interventions aimed at favoring social well-being in those engaged students who have a social impairment. Therefore, in line with Loscalzo and Giannini [8], we recommend implementing school-based interventions to improve time management skills in students, in order to teach them how to balance academic success and good social relations.

Besides the path analysis model, we conducted MANOVAs to explore whether students with high Studyholism/Study Engagement differ from those with low levels of these study behaviors. We found that students with high Studyholism score higher than those with low Studyholism on social anxiety, negative interpretations in social situations, belief in negative interpretations in social situations, and all the types of interpretation in non-social situations (but not on belief in negative interpretations). Therefore, the path analysis highlighted that the tendency to interpret non-social situations negatively and neutrally are predictors of Studyholism (or they are potential mechanisms implied in the development and maintenance of Studyholism). MANOVAs, instead, showed that high Studyholism (as the independent variable in this analysis) is associated with a higher tendency to have negative interpretations (and believe in them) in social situations. Since students with high Studyholism also have higher social anxiety, we speculate that the evidence of a negative interpretation bias for social situations in students with high Studyholism is due to their elevated social anxiety, which is associated with this type of interpretation bias [20,21,25]. We also found that adolescents with high Studyholism have a higher tendency to have all possible interpretations (i.e., positive, negative, and neutral) popping up in their mind when facing non-social situations, despite not differing from their peers in the probability of thinking that the negative interpretation is the most believable. Hence, we speculate that this might be explained by the obsessive nature of Studyholism, which could lead students to overthink and ruminate about ambiguous non-social situations (including academic results) that happened (or that are going to happen) to them, taking into account all the possible explanations and not being able to reach a firm conclusion about how an event should be interpreted. Therefore, we advocate that this might support the theorization of Studyholism as an OCD-related disorder (or as an internalizing disorder). However, future studies should deepen the analysis of the interpretation bias, especially regarding academic performance, in Studyholics.

Concerning Study Engagement, the MANOVA indicated that students with high levels score higher on social anxiety, positive and neutral interpretations in social situations, and all the types of interpretation in non-social situations (except for the belief in negative interpretations). Hence, it is confirmed that some engaged students have high social anxiety, and this suggests that those at-risk for high social anxiety are the ones with very high levels of Study Engagement: their heavy study investment might be an over-reaction to their social fears. The overstudying behavior might be implemented to avoid social embarrassment as a consequence of bad grades or the inability to answer a question. However, the general positive value of high Study Engagement is confirmed thanks to a higher tendency for positive and neutral interpretations in social situations and a higher tendency for all the types of interpretation in non-social situations, without preferring the negative one. However, on this last point concerning non-social situations, it should be noted that this is the same result we found for Studyholism. We suggest that a possible explanation is that high Studyholism and high Study Engagement might be co-present in some students [1]; therefore, there might be some similar features in students with high levels of the two types of heavy study investment. Moreover, a previous study found that paranoid ideation predicts Study Engagement [15]; hence, we could suggest that an overthinking/rumination attitude might also be present in students with high Study Engagement.

On the differences between Engaged and Disengaged Studyholics, Mann-Withney tests highlighted only a statistically significant difference: Engaged Studyholics, in social situations, have a higher tendency for positive interpretations than Disengaged Studyholics. Hence, as suggested by Loscalzo and Giannini [1], it is critical to distinguish between the two types of Studyholic since there might be some differences to consider for preventive and clinical interventions. However, other studies should further analyze this result as we have a higher percentage of Engaged Studyholics (5.7%) than Disengaged Studyholics (0.9%); most importantly, we have just five students in the Disengaged Studyholics group. Hence, our results should be read cautiously.

We also explored whether Studyholism and Study Engagement levels differ based on the type of school and school year. We found that students from the professional school have lower Studyholism and Study Engagement levels than students from the three types of high school, while there are no differences between the different high schools. Hence, in line with Loscalzo and Giannini’s [1] supposition and Loscalzo et al.’s [19] findings, we observed that high school students have higher Studyholism and Study Engagement than professional school students. Generally, high schools press more toward high study investment than technical and professional schools. Therefore, Loscalzo and Giannini [1] suggested that this might lead to higher Studyholism, and we speculate that this could also apply to Study Engagement. However, Loscalzo et al. [19] did not find a difference in Studyholism levels between technical and high schools. Hence, it seems that high schools are the type of school where Studyholism is more spread, at least based on the currently available findings. In addition, Loscalzo [9] did not find that type of school is a predictor of Studyholism and Study Engagement. Therefore, we might venture that it is not the type of school to foster Studyholism. Instead, we suggest that students characterized by high Study Engagement and Studyholism tend to choose high schools rather than technical and professional schools to satisfy their need for intense studying. Future studies should include students from technical schools (not represented in our study) to deepen the analysis of heavy study investment in this type of school. From a practical point of view, our results suggest that preventive interventions aimed at detecting students at-risk of high Studyholism (and Engaged students at-risk for social anxiety and social impairment) should be primarily implemented in high schools.

Finally, the last MANOVA we performed showed that students in their last year have higher Studyholism than peers of all the other years (except for second-year students). Our results align with the speculation that when students are close to their final exam (fifth year), they could feel more pressured to overstudy than in previous years [1]. However, this contrasts with Loscalzo et al.’s study [19], which did not find a difference in Studyholism but found it for Study Engagement (first-year students have higher Study Engagement than third-year students). Given that there are few studies on adolescents, we recommend that future studies investigate Studyholism (and Study Engagement) in adolescents, also aiming to provide further insight into the relationship between these types of heavy study investment and the school year. In fact, our study has a different representation of students compared to Loscalzo et al. [19]: we have a higher percentage of students from professional schools and no students from technical schools; however, both studies have about half of the students from high school. Based on the available evidence, we recommend implementing preventive interventions since the first year of the second-grade secondary school and, possibly, even at lower school levels (including primary school).

Among the main limitations of the study, there is a lack of students from technical schools; hence, the sample is not representative of all Italian types of second-grade secondary school. In fact, the Italian school system foresees three types of school for adolescents: high school, technical school, and professional schools. Technical schools address training regarding fundamental areas for the country’s economic and productive development, while professional schools implement job-related laboratory activities [46,47,48]. Moreover, all the adolescents attended a school in a city located in Central Tuscany, offering different curricula options (i.e., a professional school and three high schools), further reducing the results’ generalizability. Also, given the high prevalence of Engaged Studyholics and the low prevalence of Disengaged Studyholics, the differences between the two types of Studyholic have been analyzed using non-parametric analyses, and the group of Disengaged Studyholics is tiny. Hence, the related results should be read cautiously.

Despite these limitations, the current research has the merit of having analyzed Studyholism, a new potential clinical condition, in a sample (quite balanced for gender) of adolescents. There is little research published on Studyholism, especially in adolescents; hence, the findings presented by this study provide further insight on this new construct, with important implications for its theorization and preventive and clinical interventions. Furthermore, it highlighted that Study Engagement—even if generally associated with positive outcomes—should receive clinical attention, especially when present at very high levels and if associated with social impairment, since this might represent a coping strategy for social fears.

## 5. Conclusions

Studyholism, or obsession toward study, is a new potential clinical condition associated with negative academic, physical, and psychological outcomes, in contrast with Study Engagement, which predicts positive outcomes. However, previous studies showed that both Studyholism and Study Engagement predict social impairment. Therefore, we designed this study to detect some psychological features that might be addressed to reduce Studyholism and the social well-being impairment that might characterize Studyholics and Engaged students. Hence, we selected social anxiety and interpretation bias as the variables to be analyzed in adolescents.

Among the main findings, social anxiety is a positive predictor of Studyholism (to a greater extent) and Study Engagement. These results support the conceptualization of Studyholism as an OCD-related disorder or, more generally, as an internalizing disorder. However, as highlighted in our previous papers, the literature about problematic overstudying is still too scant to reach any firm conclusion. Moreover, the results indicate that socially anxious adolescents should be screened for Studyholism, and social anxiety treatments should be tested for efficacy in reducing Studyholism. Moreover, we recommend screening Engaged students (especially those with high social impairment) for social anxiety.

In terms of the interpretation bias, we found that Studyholism is predicted by the tendency to interpret non-social situations negatively and neutrally, in line with its theorization as a potential clinical disorder. Study Engagement is predicted by the tendency to interpret non-social situations positively and (even if marginally) by the tendency to believe less in negative interpretations in social situations. Since the interpretation bias in social situations (which is a feature of socially anxious adolescents) does not predict Studyholism, we speculate that social anxiety and Studyholism are two different diagnoses, even if social anxiety might fuel the development of Studyholism. Finally, we suggest that interventions aimed at reducing Studyholism should address the interpretation style for non-social situations to decrease the tendency to interpret them negatively or neutrally while promoting the tendency to interpret these situations positively. Instead, the interpretation bias does not seem a proper target for interventions aimed at favoring social well-being in engaged students. Hence, we recommend implementing time management skills to balance academic success and social life towards this aim.

Finally, the currently available results about differences in Studyholism and Study Engagement levels based on the type of school allow suggesting that these study behaviors are more spread in high schools, probably because Engaged and Studyholics students tend to choose high schools, where they can satisfy their need for intense studying. Hence, even if preventive interventions should be implemented across all schools, high schools should be the primary target.

In conclusion, the present study has the main merit of having analyzed Studyholism, a new potential clinical condition, in adolescents, with critical theoretical and practical implications. It also highlighted that Study Engagement, even if generally associated with positive outcomes, should receive attention, especially when present at very high levels and if associated with social impairment, since this might represent a coping strategy for social fears. Therefore, we suggest that future studies analyze further Studyholism and Study Engagement in adolescents, including the role of interpretation bias (focusing on situations related to school performance) and comprising adolescents attending all the types of school (i.e., professional, technical, and high school). Finally, we believe that our findings might have a critical value also concerning the mental health issues associated with the current COVID-19 pandemic. We speculate that the forced social isolation lived by adolescents in the last years might have heightened social anxiety levels (especially in those who were at-risk for social anxiety); therefore, heavy study investment might constitute a strategy to cope with the distress caused by the health outbreak and a means to keep avoiding social contacts.

## Figures and Tables

**Table 1:** Descriptive statistics of the variables in the model (*n* = 541).

Variable	Range	M (DS)	Skeweness	Kurtosis
Studyholism	4–20	10.50 (4.69)	0.33	−0.90
Study Engagement	4–20	11.61 (4.55)	0.04	−0.94
Social Anxiety	0–66	23.95 (14.81)	0.38	−0.59
Social–Positive Interpretations	1–4.80	2.54 (0.75)	0.13	−0.28
Social–Negative Interpretations	1–5	2.77 (0.87)	0.30	−0.28
Social–Neutral Interpretations	1–5	3.29 (0.75)	−0.35	0.21
Social–Belief in Negative Interpretations	1.20–3	2.05 (0.35)	0.20	−0.10
Non-Social–Positive Interpretations	1–5	3.27 (0.71)	−0.35	0.30
Non-Social–Negative Interpretations	1–5	2.79 (0.71)	0.13	−0.05
Non-Social–Neutral Interpretations	1–4.60	2.75 (0.69)	−0.05	−0.28
Non-Social–Belief in Negative Interpretations	1–2.80	1.80 (0.35)	0.28	−0.02

**Table 2:** Zero-order correlations for study variables (*n* = 541).

Variable	1	2	3	4	5	6	7	8	9	10	11
1.Studyholism	-										
2.Study Engagement	0.54 ***	-									
3.Social Anxiety	0.42 ***	0.18 ***	-								
4.Social–Positive Interpretations	−0.03	0.15 ***	−0.16 ***	-							
5.Social–Negative Interpretations	0.24 ***	0.08	0.44 ***	−0.14 ***	-						
6.Social–Neutral Interpretations	0.10 *	0.18 ***	−0.01	0.24 ***	0.05	-					
7.Social–Belief in Negative Interpretations	0.12 **	−0.10 *	0.24 ***	−0.46 ***	0.52 ***	−0.11 *	-				
8.Non-Social–Positive Interpretations	0.11 **	0.22 ***	0.02	0.32 ***	0.16 ***	0.45 ***	−0.09 *	-			
9.Non-Social–Negative Interpretations	0.22 ***	0.14 ***	0.17 ***	0.19 ***	0.42 ***	0.19 ***	0.12 **	0.19 ***	-		
10.Non-Social–Neutral Interpretations	0.17 ***	0.13 **	0.02	0.35 ***	0.18 ***	0.33 ***	−0.08	0.32 ***	0.16 ***	-	
11.Non-Social–Belief in Negative Interpretations	0.05	−0.01	0.09 *	−0.11 **	0.12 **	−0.18 ***	0.13 **	−0.39 ***	0.35 ***	−0.07	-

*Note*. *** *p* ≤ 0.001; ** *p* ≤ 0.01; * *p* < 0.05.

**Table 3:** Standardized path weights of the path analysis model (*n* = 541).

Dependent Variable	Predictor	*β*	*p*
Studyholism	Social–Positive Interpretations	−0.07	n.s.
	Social–Negative Interpretations	−0.06	n.s.
	Social–Neutral Interpretations	0.02	n.s.
	Social–Belief in Negative Interpretations	0.02	n.s.
	Non-Social–Positive Interpretations	0.05	n.s.
	Non-Social–Negative Interpretations	0.16	0.002
	Non-Social–Neutral Interpretations	0.15	<0.001
	Non-Social–Belief in Negative Interpretations	−0.01	n.s.
	Social Anxiety	0.40	<0.001
Study Engagament	Social–Positive Interpretations	0.05	n.s.
	Social–Negative Interpretations	−0.01	n.s.
	Social–Neutral Interpretations	0.08	n.s.
	Social–Belief in Negative Interpretations	−0.11	0.049
	Non-Social–Positive Interpretations	0.17	0.002
	Non-Social–Negative Interpretations	0.04	n.s.
	Non-Social–Neutral Interpretations	0.02	n.s.
	Non-Social–Belief in Negative Interpretations	0.06	n.s.
	Social Anxiety	0.20	<0.001

**Table 4:** Follow-up ANOVAs. AIBQ scales and social anxiety by low and high Studyholism (SH) and Study Engagement (SE).

Variable		Level	*n*	*M (SD)*	*F* ^§^	*p*	*Partial η* ^2^
S–Positive Interpretations	SH	Low	169	2.49 (0.79)	0.11	n.s.	0.000
		High	72	2.45 (0.70)			
		Total	241	2.48 (0.76)			
	SE	Low	182	2.38 (0.76)	7.48	0.007	0.03
		High	66	2.67 (0.73)			
		Total	248	2.46 (0.76)			
S–Negative Interpretations	SH	Low	169	2.51 (0.85)	25.66	<0.001	0.10
		High	72	3.12 (0.87)			
		Total	241	2.69 (0.90)			
	SE	Low	182	2.69 (0.92)	1.63	n.s.	0.01
		High	66	2.86 (0.90)			
		Total	248	2.74 (0.92)			
S–Neutral Interpretations	SH	Low	169	3.22 (0.84)	3.74	n.s.	0.02
		High	72	3.44 (0.70)			
		Total	241	3.29 (0.80)			
	SE	Low	182	3.16 (0.86)	13.60	<0.001	0.05
		High	66	3.58 (0.56)			
		Total	248	3.28 (0.81)			
S–Belief in Negative Interp.	SH	Low	169	2.02 (0.36)	5.83	0.017	0.02
		High	72	2.14 (0.31)			
		Total	241	2.05 (0.35)			
	SE	Low	182	2.07 (0.37)	1.78	n.s.	0.01
		High	66	2.01 (0.31)			
		Total	248	2.06 (0.35)			
NS–Positive Interpretations	SH	Low	169	3.16 (0.75)	5.87	0.016	0.02
		High	72	3.41 (0.75)			
		Total	241	3.23 (0.76)			
	SE	Low	182	3.11 (0.74)	19.35	<0.001	0.07
		High	66	3.55 (0.60)			
		Total	248	3.23 (0.73)			
NS–Negative Interpretations	SH	Low	169	2.60 (0.72)	20.60	<0.001	0.08
		High	72	3.06 (0.69)			
		Total	241	2.73 (0.74)			
	SE	Low	182	2.64 (0.79)	4.32	0.039	0.02
		High	66	2.87 (0.65)			
		Total	248	2.70 (0.76)			
NS–Neutral Interpretations	SH	Low	169	2.58 (0.73)	17.77	<0.001	0.07
		High	72	2.99 (0.61)			
		Total	241	2.70 (0.72)			
	SE	Low	182	2.67 (0.74)	3.92	0.049	0.02
		High	66	2.87 (0.67)			
		Total	248	2.72 (0.72)			
NS–Belief in Negative Interp.	SH	Low	169	1.80 (0.33)	0.33	n.s.	0.001
		High	72	1.82 (0.39)			
		Total	241	1.80 (0.35)			
	SE	Low	182	1.80 (0.36)	0.10	n.s.	0.000
		High	66	1.81 (0.33)			
		Total	248	1.80 (0.35)			
Social Anxiety	SH	Low	169	16.19 (12.07)	86.97	<0.001	0.27
		High	72	32.82 (13.98)			
		Total	241	21.16 (14.77)			
	SE	Low	182	20.75 (15.37)	7.68	0.006	0.03
		High	66	26.79 (14.57)			
		Total	248	22.36 (15.36)			

*Note*. AIBQ = Adolescents’ Interpretation and Belief Questionnaire; S = Social; NS = Non-Social; ^§^ = for Studyholism, df = 1, 239; for Study Engagement, df = 1, 246.

**Table 5:** Mann-Whitney tests for Studyholism antecedents and outcomes by Studyholic type.

Dependent Variable	*U*	*Z*	*p*	*r*	Type of Studyholic	*Median*	*n*
S–Positive Interpretations	32.00	−2.11	0.037	−0.35	Engaged	2.60	31
					Disengaged	1.80	5
					Total	2.60	36
S–Negative Interpretations	55.50	−1.01	n.s.	−0.17	Engaged	3.00	31
					Disengaged	3.20	5
					Total	3.00	36
S–Neutral Interpretations	43.00	−1.59	n.s.	−0.27	Engaged	3.60	31
					Disengaged	3.00	5
					Total	3.50	36
S–Belief in Negative Interp.	51.00	−1.24	n.s.	−0.21	Engaged	2.00	31
					Disengaged	2.60	5
					Total	2.00	36
NS–Positive Interpretations	47.00	−1.41	n.s.	−0.24	Engaged	3.60	31
					Disengaged	3.00	5
					Total	3.50	36
NS–Negative Interpretations	46.00	−1.45	n.s.	−0.24	Engaged	3.00	31
					Disengaged	4.00	5
					Total	3.00	36
NS–Neutral Interpretations	60.00	−0.81	n.s.	−0.14	Engaged	3.40	31
					Disengaged	3.00	5
					Total	3.20	36
NS–Belief in Negative Interp.	69.50	−0.37	n.s.	−0.06	Engaged	1.80	31
					Disengaged	2.00	5
					Total	1.80	36
Social anxiety	56.50	−0.96	n.s.	−0.16	Engaged	31.00	31
					Disengaged	39.00	5
					Total	32.00	36

*Note*. S = Social; NS = Non-Social; Interp. = Interpretations.

## Data Availability

The dataset is available, upon reasonable request, and for research purposes only, by writing to the corresponding author.
